# Recent advances in understanding and treating priapism

**DOI:** 10.12703/r/11-23

**Published:** 2022-08-26

**Authors:** Hussain M Alnajjar, Asif Muneer

**Affiliations:** 1 Department of Urology, Institute of Andrology, University College London Hospitals NHS Trust, London, UK; 2 Division of Surgery and Interventional Science, University College London, London, UK; 3 NIHR Biomedical Research Centre, University College London Hospitals NHS Trust, London, UK

**Keywords:** priapism, ischaemic, non-ischaemic, stuttering, shunt, penile prosthesis, penile doppler, erectile dysfunction

## Abstract

Priapism is a rare condition that can lead to long-term erectile dysfunction if left untreated. It is one of the few urological emergencies that require prompt medical intervention. Priapism refers to a penile erection that lasts for more than 4 hours and is unrelated to sexual stimulation or orgasm. The aims of immediate intervention for ischaemic priapism are to resolve the painful erection and preserve the cavernosal smooth muscle function. The aim of this review is to evaluate the latest advances in the management of priapism. Despite the continuous challenge in providing an optimal treatment for this rare urological condition, our understanding and management of it have been advanced by decades of clinical and basic science research. Proximal shunts (Quackels or Grayhack) are no longer routinely performed. Distal shunt procedures are currently the most commonly used techniques. A novel penoscrotal decompression technique has recently been described. Ischaemic priapism can be managed conservatively in most cases with the preservation of erectile function. In cases where ischaemic priapism has persisted for more than 36 hours, the majority will develop erectile dysfunction. Early penile prosthesis with thorough patient counselling should be considered in such cases. In some cases of long-standing non-ischaemic priapism, patients can develop fibrosis within the distal corpora, and, therefore, early treatment with super-selective embolisation is required to prevent this.

## Introduction

Priapism is a rare condition and a urological emergency, with an estimated incidence of 1.5 cases per 100,000 men^1^. Ischaemic priapism is by far the commonest subtype and accounts for more than 95% of all priapism episodes^2^. Any delay in the presentation or failure to intervene in a timely fashion can lead to irreversible erectile dysfunction, penile shortening, loss of penile girth, and penile curvature. Despite over two decades of both clinical and basic science research, the pathophysiology of priapism is still not yet fully understood. However, various studies have suggested dysregulation of the normal neurovascular and veno-occlusive mechanisms which mediate physiological erection. Any disruption in this mechanism will lead to an unwanted prolonged erection^3^. There are three subtypes of priapism: ischaemic, non-ischaemic, and stuttering priapism, also known as recurrent ischaemic priapism.

Ischaemic priapism is the commonest subtype. The term ischaemic priapism is preferable to low-flow priapism, particularly when interpreting penile doppler imaging in the acute setting, as the term ‘ischaemic’ emphasises a degree of clinical urgency in the management of this urological emergency^4^. Non-ischaemic priapism or high-flow priapism involves unregulated oxygenated arterial blood flow into the penis. As there is an absence of ischaemia, this is unlikely to result in smooth muscle necrosis. Idiopathic ischaemic priapism is common, although other risk factors include haematological dyscrasias, medications, illicit drugs, neurological disorders, and toxic infections, and rarely it can be secondary to malignant disease due to either pelvic malignancy or secondary infiltration into the corpora via haematogenous spread^5,6^, whereas penile and perineal trauma are common causes of non-ischaemic priapism.

In 2018, the British Association of Urological Surgeons published a consensus statement with clear recommendations on the assessment and management of acute priapism; the statement provides an algorithm to be used in non-specialist centres to undertake prompt treatment^4^.

## Clinical evaluation and investigations

Classically, a clinical history and examination are key to the diagnosis of priapism. Often the presentation is delayed and commonly past the critical 4-hour time frame, whereby cavernosal smooth muscle necrosis sets in. Patients presenting with priapism lasting longer than 4 hours will require immediate medical intervention.

In contrast, non-ischaemic priapism often has a history of perineal or penile injury, which may have occurred several days to weeks before the development of priapism. A recent review of malignant priapism reported that solid tumour invasion — both primary and secondary — and haematological malignancies represent the key aetiologies of malignant priapism, and aggressive treatment is needed^6^. Cavernosal smooth muscle function can be preserved if intracavernosal phenylephrine is promptly administered or distal shunts are performed; however, the prognosis is often poor, and subsequent adjuvant chemotherapy or radiotherapy is often required^6^. However, with extensive infiltration of the corpora, this intervention is unlikely to resolve the priapism and a palliative penectomy can be considered for pain control if adjuvant treatments fail.

In ischaemic priapism, the typical presenting signs are severe penile pain and persistent unwanted hard erection, and so distinguishing the subtypes (ischaemic vs. non-ischaemic) is important. Cavernosal blood aspiration for analysis will confirm the presence of hypoxia and acidosis in ischaemic cases. Blood and urine samples must be obtained from the patient to screen for haemoglobinopathies, autoimmune conditions, and toxicology. Performing an abdominal and chest computed tomography to investigate for an underlying cause in unexplained cases is warranted. Ensuring early input from haematology at an early stage is recommended where a haematological disorder is thought to be the predisposing factor (e.g., sickle cell anaemia). Sickle cell priapism is caused by sickled erythrocytes obstructing venous outflow and can lead to severe penile pain and permanent erectile dysfunction^7^. Case reports of ischaemic priapism have been described in the literature in adult patients with severe acute respiratory syndrome coronavirus 2 (SARS-CoV-2) infection. Infection with SARS-CoV-2 has been associated with systemic inflammatory response syndrome resulting in a procoagulant state and increased incidence of arterial and venous thromboembolic events, linking both conditions to a theoretically similar pathophysiology^8^ (see Table 1).

**Table 1.** Causes of priapism.

Priapism subtypes	Causes
Ischaemic priapism	• Idiopathic, haemoglobinopathies (e.g., sickle cell anaemia, thalassemia and leukaemia) • Illicit drugs (e.g., cocaine and cannabis) • Medications^11^ (e.g., antipsychotics, antidepressants, intracavernosal PGE-1 injections, PDE-5 inhibitors, anticoagulants and alpha-blockers) • Pelvic malignancy, neurological disorders (e.g., cauda equina and spinal cord injury) • Infections (e.g., SARS-CoV-2)^12^ • Toxins (e.g., black widow spider bite, scorpion sting and carbon monoxide poisoning) • Metabolic disorders (e.g., amyloidosis)
Non-ischaemic priapism	Trauma to penis or perineum and treatment of ischaemic priapism (by creating a *de novo* fistula during aspiration and injection of sympathomimetics)

PDE-5, phosphodiesterase 5; PGE-1, prostaglandin E1; SARS-CoV-2, severe acute respiratory syndrome coronavirus 2. Modified from Muneer *et al*.^13^ under the terms of the Creative Commons Attribution 4.0 International license (CC-BY 4.0).

## Advances in radiological imaging in priapism

The diagnosis can be confirmed by a colour penile Doppler ultrasonography, where the blood flow within the cavernosal arteries and corpus cavernosum can be evaluated. Colour penile Doppler imaging can further establish a reduced or absent blood flow of the cavernosal arteries with impaired perfusion of the corpus cavernosum in cases of prolonged ischaemic priapism. In atypical cases or where there is a diagnostic uncertainty, a colour penile Doppler ultrasound may confirm the diagnosis and detect an arterio-cavernous fistula^9^.

However, aberrant blood flow may form in segments of the corpus cavernosum if a prior corporal blood aspiration had taken place; this phenomenon can lead to difficulty in interpreting a penile Doppler study^9,10^.

A 2017 study showed that a peak systolic velocity (PSV) of less than 50 cm/s and a mean velocity less than 6.5 cm/s were predictive of ischaemic priapism (pre-shunt); however, patients with ischaemic priapism showed a PSV of more than 22 cm/s but had a diastolic reversal and therefore low net perfusion^9^.

**Figure 1.**
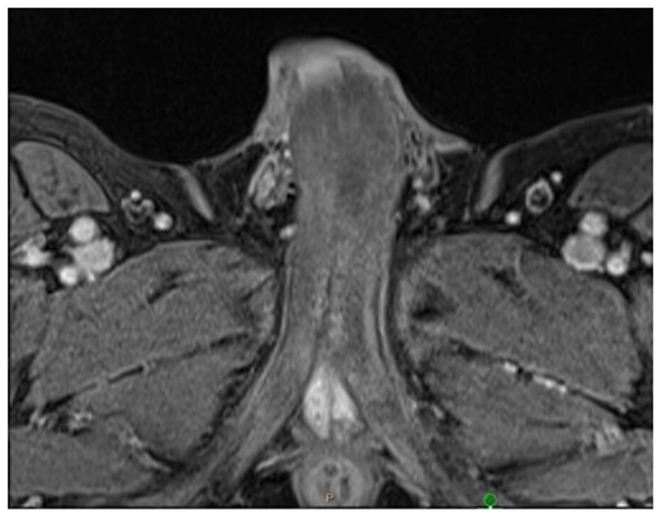
Axial magnetic resonance image illustrating poor enhancement areas within both corpora cavernosa as a result of necrosis and early fibrosis.

Furthermore, the use of magnetic resonance imaging (MRI), colour ultrasound Doppler imaging, and cavernosal smooth muscle biopsies has revolutionised the current practice in the management of acute, persistent ischaemic priapism^14^. Gadolinium-enhanced high-definition T1/T2-weighted MRI of the penis (Figure 1) has 100% sensitivity in detecting cavernosal smooth muscle necrosis and fibrosis^15^.

## Management of priapism

Management of priapism focuses on four principles: controlling the acute pain, resolving the acute event, preserving erectile function, and preventing the risk of future recurrences. Conservative management is the mainstay in the treatment of early stages of priapism. This includes pain management, strenuous physical activity, ejaculation, and application of ice packs to the upper-inner thigh may induce detumescence by sympathomimetic response; if conservative manoeuvres fail, then a stepwise approach is used to resolve the acute event^4^. Those who are refractory to these interventions or who have prolonged priapism may benefit from the early placement of a penile prosthesis in the ischaemic cases or selective arterial embolisation in non-ischaemic priapism.

### A. Cavernosal decompression

**Figure 2.**
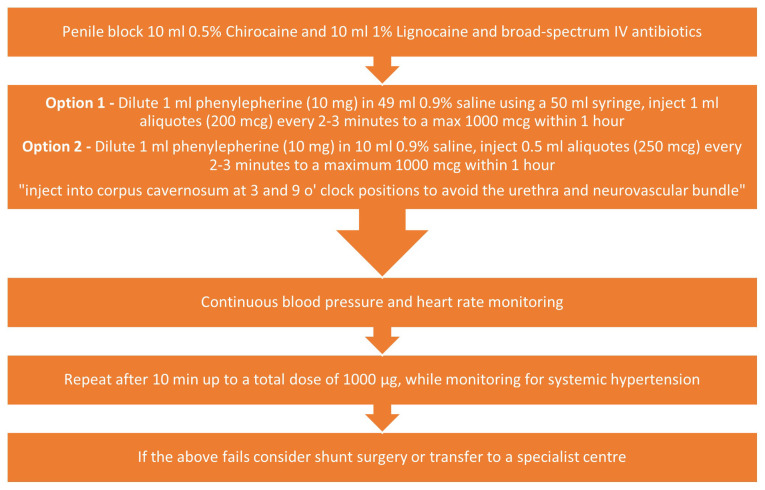
Administration of phenylephrine.

Once a penile block has been performed, a 19-gauge butterfly needle is inserted into the corpora via the glans penis or directly into the corpora (penile shaft at the 2 or 10 o’clock positions). Up to 50 ml of blood should be aspirated from the corpus cavernosum, and a sample is analysed by a blood gas machine. If this fails, instillation of α agonists (e.g., phenylephrine) is the next step (Figure 2). This leads to an increased smooth muscle tone of the corpora cavernosa, which promotes detumescence. Other adrenergic agonists such as metaraminol and adrenaline have been suggested in the American Urological Association guidelines but are used less frequently. Continuous monitoring of the blood pressure and heart rate is required, as α agonists may precipitate a cardiovascular event. Corporal blood aspiration and instillation of α agonists are less likely to respond in priapism episodes lasting more than 24 hours due to irreversible smooth muscle damage. Erectile function recovery is seen in about 50% of ischaemic priapism cases if reversed within 24 hours of onset, whereas a duration of more than 36 hours is invariably associated with a degree of corporal fibrosis and erectile dysfunction^16^. Both *in vitro* and *in vivo* studies have shown that the presence of hypoxia and glucopenia together leads to cavernosal smooth muscle dysfunction and that smooth muscle necrosis starts at 4 hours^17^.

### B. Surgical shunts (corporo-glanular)

If all of the above approaches have failed, a surgical shunt is the next step. The shunt enables drainage of ischaemic blood from the corpora cavernosa to either the corpus spongiosum or the saphenous vein; however, proximal shunts (Quackels or Grayhack) are not routinely performed. Currently, distal shunt procedures are the most commonly used as they are easier to perform and seem to have comparable detumescence and potency rates^18^.

**Figure 3.**
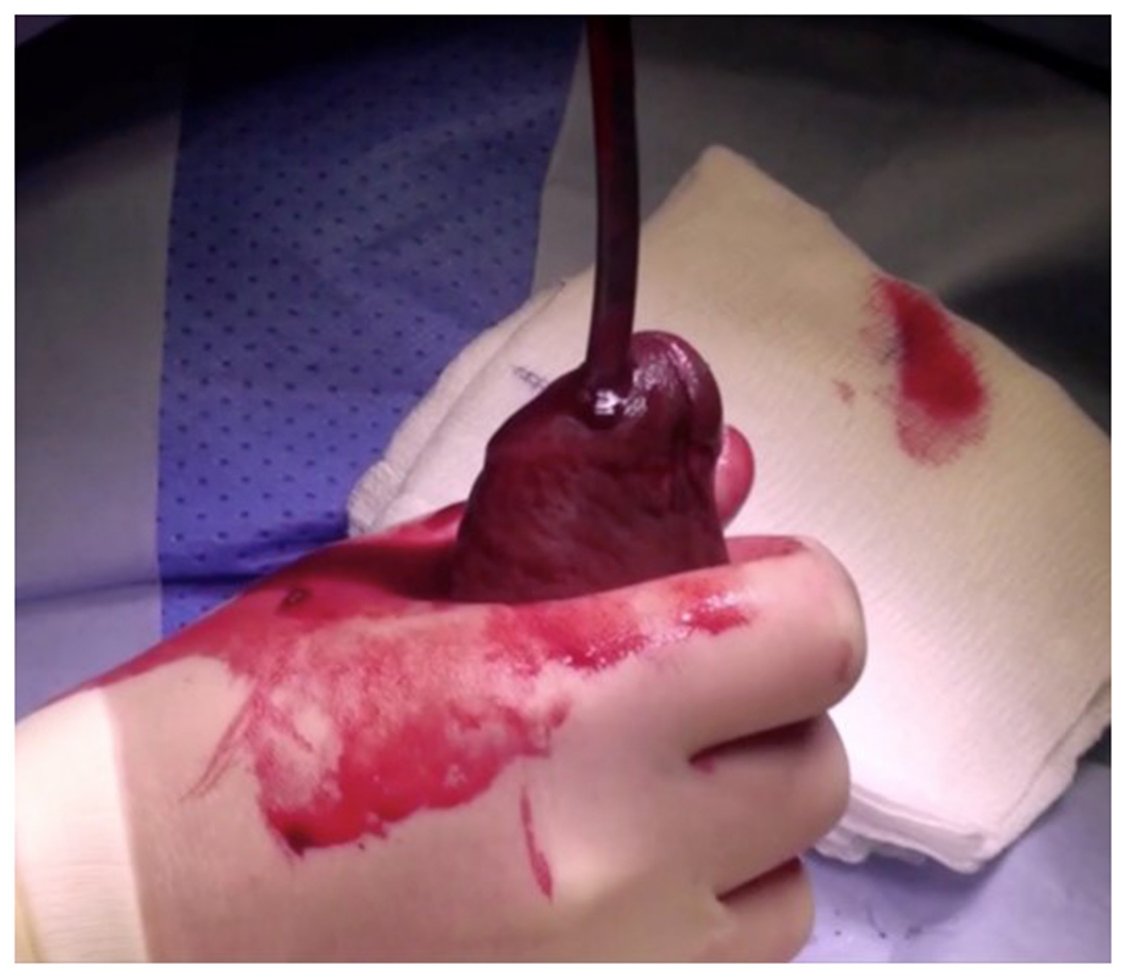
Corporal tunnelling (snake manoeuvre).

More recently, the T-shunt has been used widely, aiming to create superior communication between the glans penis and the distal end of the corpora. The technique involves the insertion of a No. 11 scalpel blade through the glans penis into the ipsilateral corpus cavernosum followed by a 90° rotation laterally away from the urethra to create a fistula^19^. The procedure can be repeated on the contralateral side in cases where detumescence has not been achieved (TT shunt). A corporal tunnelling manoeuvre (snake manoeuvre) may be attempted if the TT shunt has failed (Figure 3); this technique aims to drain the ischaemic blood from the proximal part of the corpora cavernosa by advancing a 7 or 8 Hegar dilator through the previous T-shunt windows proximally to allow the ischaemic blood to drain^20^.

A study reported that the success of the T-shunt with snake tunnelling is dependent on the duration of priapism^21^. Erectile dysfunction is still present in about 50% of patients if the procedure was performed within 24 hours of the priapism. In priapism cases lasting longer than 48 hours, the technique usually fails, and all patients develop erectile dysfunction due to smooth muscle necrosis followed by fibrosis^21^.

More recently, a novel penoscrotal decompression (PSD) technique for refractory ischaemic priapism was described^22^. The technique involves a penoscrotal incision followed by a corporotomy to allow drainage of the ischaemic blood. This glans-sparing corporal decompression technique aims to allow spontaneous recovery of erectile function and therefore reduce the need for implantation of a penile prosthesis^22^. A recent retrospective multicentre study reported on 10 patients undergoing unilateral PSD, of whom two (20%) had a recurrence of priapism^23^. A further 15 patients underwent primary bilateral PSD; none had priapism recurrence. Subsequently, nine (60%) reported spontaneous erectile function adequate for penetration, and six (40%) reported erectile dysfunction. The study established that PSD is a simple, safe, and highly effective treatment for refractory ischaemic priapism; however, further larger prospective studies and randomised controlled trials are required to confirm these findings^23^.

### C. Implantation of penile prosthesis in the acute setting

Failure to achieve detumescence following the above treatment modalities will lead to irreversible smooth muscle necrosis and refractory long-term erectile dysfunction. An erection lasting 24 to 48 hours inevitably leads to permanent smooth muscle damage and subsequent fibrosis. The high rates of impotence, corporal fibrosis, and loss of penile length and girth reported in the current literature suggest that these patients benefit from immediate penile prosthesis placement in cases of refractory priapism^14^.

Because it has fewer components, acute insertion of a malleable prosthesis is associated with a lower risk of infection and is simpler to insert within the first 3 weeks of the onset of priapism. If there is any further delay, then an inflatable prosthesis should be considered at 6 to 8 weeks as there will be established cavernosal fibrosis resulting in a difficult corporal dilatation regardless of the type of device being used. At our institution and in our experience of early malleable prosthesis insertion, downsizing the malleable prosthesis by 1 to 2 cm is performed in order to reduce the risk of distal erosion and extrusion, particularly if the patient has undergone a recent T-shunt or a corporal dilatation procedure. Moreover, there should be a 2-week interval between the T-shunt procedure and the insertion of a malleable prosthesis to allow the fistula to heal. After 6 months, the malleable prosthesis can be replaced by a three-piece inflatable prosthesis, allowing upsizing of the cylinders^24^.

In severely fibrotic and challenging cases, penile prosthesis implantation with simultaneous total corporal reconstruction seems to offer reasonable outcomes. Nevertheless, complication rates are significantly higher because of severe fibrosis, and consequently, patient counselling and discussions on expectations are crucial^25^. Several other techniques (e.g., corporal excavation, narrow prostheses, and multiple corporal incisions) have been employed to reduce complications and improve surgical outcomes^26^. Patients should be assessed on a case-by-case basis and counselled on the risks and benefits of early versus delayed insertion of a penile prosthesis. Currently, there is no absolute evidence regarding the ideal device or timing of implantation. However, there are well-established reports of the advantages and disadvantages of malleable versus inflatable prostheses and of acute versus delayed implantation^27^.

Moreover, the current guidelines of the American Urological Association/Sexual Medicine Society of North America support the concept of early placement of a penile prosthesis in untreated acute ischaemic priapism cases greater than 36 hours or in those who are refractory to shunting procedures, with or without tunnelling. The guidelines also state that clinicians should discuss the risks and benefits of the early versus delayed approach if early placement of a penile prosthesis is being considered^28^. After many years of translational research, it has been established that time-dependent irreversible changes are inevitable in refractory cases; therefore, early placement of penile prosthesis has become the standard of care in specialist centres^29^.

## Stuttering priapism

Stuttering priapism is the least common subtype and is usually a self-limiting condition. It generally lasts less than 3 to 4 hours per episode. However, it has the propensity to develop into full ischaemic priapism in 30% of cases. The condition shares its aetiologies with ischaemic priapism. The management of stuttering priapism aims primarily to prevent recurrence rather than the resolution of spontaneous attacks^30^.

A recent small study reported daily dutasteride therapy as a promising treatment option to reduce the frequency and severity of the painful episodes without significant side effects^31^.

Daily low-dose phosphodiesterase 5 (PDE-5) inhibitor treatment in a small series of patients prevented recurrent priapism while preserving normal erectile function^32^. A recent cohort study evaluated the efficacy of various treatments in patients with sickle cell-related stuttering priapism^33^. The study demonstrated that hydroxyurea and automated exchange transfusion were the most effective treatment options, but that hormone manipulation and α-agonist (e.g., etilefrine) therapies were effective in both idiopathic stuttering priapism and sickle cell-related stuttering priapism cases^33^.

Another rare entity is sleep-related painful erection (SRPE), where patients report bothersome nocturnal erections that wake them up and result in poor-quality sleep and daytime somnolence. Abnormal sleep architecture with rapid eye movement (REM) awakenings and significantly more periodic limb movements have been reported in patients with SRPE, suggesting a central (sleep-related) cause^34^. Baclofen seems to be the most effective treatment in this uncommon group of patients.

## Non-ischaemic priapism

Non-ischaemic priapism results in persistent partial penile tumescence because of the high flow of arterial blood into the corpora as a result of trauma to the penis or perineum. Non-ischaemic priapism is much less common than the ischaemic subtype and does not require urgent surgical intervention. The flow of oxygenated blood within the corpora and the lack of severe penile pain permit non-ischaemic cases to be initially managed conservatively. Hence, at the outset, it is vital to have an accurate diagnosis which can be confirmed by Doppler ultrasonography. Following a period of conservative management, which necessitates regular outpatient clinical review, diagnostic angiography combined with super-selective embolisation of the fistula can be performed with various agents or microcoils. The literature quotes erectile dysfunction rates as low as 5% when using temporary agents and 39% with permanent agents. However, a recent study demonstrated that erectile dysfunction rates were higher with temporary agents than with permanent agents (17–33% vs. 8–17%)^35^. Furthermore, the choice of the embolic agent seems to be crucial and should be tailored to each patient^36^.

In some cases of long-standing non-ischaemic priapism, patients can develop fibrosis within the distal corpora, and therefore, early treatment with super-selective embolisation is required to prevent this. Patients with distal flaccidity and fibrosis within the distal corpus cavernosum should undergo a penile MRI scan as the best imaging modality in these scenarios^37^.

## Conclusions

Despite the continuous challenge in providing optimal treatment for this rare urological condition, our understanding and management of it have been advanced by decades of clinical and basic science research. Pharmacologic advances have considerably improved the management of stuttering priapism and the outlook of resolution for patients with ischaemic priapism presenting within the first few hours of the onset, whereas the management of non-ischaemic priapism is mainly expectant and only in persistent cases a super-selective embolisation may be indicated. Distinguishing the ischaemic from the non-ischaemic state is conceivably the most important diagnostic step as it outlines the series of further interventions, including surgical shunts and early implantation of penile prosthesis in refractory cases.
